# Power(ful) Connections: Exploring the Revolving Doors Phenomenon as a Form of State-Corporate Crime

**DOI:** 10.1007/s10612-022-09626-z

**Published:** 2022-04-23

**Authors:** Mònica Pons-Hernández

**Affiliations:** Center for the Study of the Environmental Law of Tarragona (CEDAT), Universitat Rovira i Virgili, Avinguda Catalunya, 35, 43002 Tarragona, Spain

## Abstract

The ‘revolving doors’ phenomenon is the name given to the movement of individuals from public office to private companies and vice versa. It is believed that this phenomenon impacts the decision-making process to the detriment of the public interest. While it has largely been framed as a corrupt practice, this research seeks to explore it as a form of state-corporate crime by employing the case study of the Spanish government and the fossil fuel industry. The study finds that the explored case of revolving doors causes harm by driving social and economic instability and climate inaction and proposes the creation of a 'state-capture corporate crime'. Overall, the paper highlights the need to broaden the revolving doors and state-corporate crime concepts to allow its inclusion in the criminological agenda.

## Introduction

Neoliberalism is a revolutionary force that has dramatically changed the relationship between government and business over the last 30 years (Whyte 2014; Bradshaw 2015; Bernat and Whyte 2020). This political-economic theory contends that private property rights, free markets, and free trade are the best ways to achieve human well-being. In this context, the role of the government is to actively create a setting where it does not interfere with the profitability of businesses and multinational corporations (Harvey 2005; Whyte 2014). According to Whyte (2013) and Bradshaw (2015), neoliberalism intensifies the interconnectedness between the public and the private sector, encouraging close collaboration between the government and corporations. A visible manifestation of this process is the *revolving door* (Whyte 2013, 2014; Bradshaw 2015).

The practice of the revolving doors (RDs) refers to the movement of individuals between public and private employment (Zheng 2015). As Kowalewski et al. (1991) indicate, the RDs is an elongated practice in developed economies. For instance, between 1897 and 1973, an average of 76% of the Cabinet members in the US had corporate ties and 37% of the members of the British House of Commons during 1918–1935 came from corporations (Kowalewski et al. 1991). While some defend that this practice enhances the performance of states and corporations (see Kowalewski et al. 1991), it can lead to the capture of regulators by industry interests (Cortese 2011; Zheng 2015). In other words, the public actors involved regulate in favor of the corporations either to secure a post-government position in the private sector or, when moving from private to public office, because they have been socialized in the industry setting (Dal Bo 2006; Cortese 2011; Zheng 2015). Either way, this phenomenon impacts the decision-making process to the detriment of the public interest (Cortese 2011). Accordingly, defining corruption as a set of relationships and practices detrimental to the public interest (Beetham 2015), the RDs have mostly been framed as a corrupt practice (see Poynting and Whyte 2017; Rawlinson 2017; Huisman and Vande Walle 2010). However, this paper argues that the RDs are a form of state-corporate crime that requires a broader consideration of the original concept.

Within the RDs are differentiated two directions: *revolving out* and *revolving in*. Revolving out refers to the movement from the public to the private employment and revolving in refers to the transition from private to public office. RDs movements appear across economic sectors and public positions with the power to regulate, enforce, or fund corporations. For example, in the US, The Revolving Door Project (n.d.) exposes cases of individuals in the Food Industry, the Defense Industry, the Big Pharma, or the Fossil Fuel Industry, among others, coming from or going to positions in Cabinet Departments, the Executive Office or Federal Agencies. Thus, it mainly involves industries of strategic sectors and political or public roles that have the power to benefit the corporations (The Revolving Door Project n.d.). However, as Wilks-Heeg (2015), The Revolving Door Project (n.d.), and this research argue, the ‘revolving in’ needs a broader conceptualization to include the political interference of lobbying groups as a new dimension of the RDs. According to Wilks-Heeg (2015), lobby groups participate in policymaking without needing the actual move into the government, bringing a new dimension to the phenomenon.

Following all the above, the research builds on the state-corporate crime theory. State-corporate crimes are criminal or *socially injurious* acts that occur when the interests of the state and the corporate sector intersect (Kramer et al. 2002). Thus, as Bisschop et al. (2018: 887) describe, the “state-corporate crime theory takes seriously the crimes of political, social and economic elites within the context of governmental and corporate institutions”. The state-corporate crime framework includes the harms resulting from the interactions between states and corporations because elite wrongdoings do not always involve the flouting of criminal laws (Kramer and Michalowski 2006; Kramer 2013). The harm-based approach implies the refusal of limiting the understanding of crime to state-definitions of crime (Hillyard and Tombs 2017; Tombs and Hillyard 2004).

Putting the role of the state at the center of the discussion (Whyte 2014), the theory confronts the aligned interests of public and private-sector elites, whether these take the form of cooperation, collusion, or corruption (Bisschop et al. 2018). The theory differentiates between state-initiated and state-facilitated corporate crime (Kramer et al. 2002; Michalowski and Kramer 2006). While state-initiated corporate crimes occur when corporations engage in crime under the direction of the government, state-facilitated corporate crimes take place when the government fails to regulate corporate activities (Kramer et al. 2002; Michalowski and Kramer 2006). More recently, Lasslett (2014) proposed adding the concepts of corporate-initiated state crime and corporate-facilitated state crime. In this case, while corporate-initiated state crime refers to corporations employing their power to manipulate states into taking criminal actions, corporate-facilitated state crime occurs when corporations provide the means for state crime (Lasslett 2014). However, these conceptualizations are limited to describing the RDs because they define a ‘moment of rupture’ instead of focusing on the political-economic structure itself (Whyte 2014: 237).

Some countries limit the RDs practice by establishing a cooling-off period, but lobbying transparency rules, cooling-off periods, and other restrictions are proving to be insufficient to avoid the social harms arising from this practice (LaPira and Thomas 2014). Because the RDs are not a criminal offense, the harm-based approach adopted in the state-corporate crime theory allows for the inclusion of the phenomenon in the criminological agenda. A harm-based approach not only challenges the definitions of crime, but also those responsible for such crimes. It allows considering the corporate and state responsibility of harm and helps to place the accountability on structural rather than individual factors (Hillyard and Tombs 2007). By leaving the harmful actions of the powerful out of the criminal spectrum, what should be considered as state-corporate crime is not perceived as a serious crime by society despite causing injuries, illness, deaths, financial loss, and environmental destruction (Kramer et al. 2002; Michalowski and Kramer 2006). As the state and corporate actors mostly break regulatory laws, these greater crimes are perceived as less serious, less harmful, and the perpetrators less criminal.

Using a case study approach, and drawing on government and non-profit organizational documents, academic articles, and journalistic reports, this paper examines the criminogenic dimension of the RDs by focusing on how the RDs in the fossil fuel industry in Spain have resulted in social and environmental damage. To allow the wider conceptualization of the victim, this project uses a green criminology philosophical underpinning. Through this green criminology perspective, the research contemplates harms to the environment apart from those to humans. In addition, the project seeks to nourish the concept of state-corporate crime. Because the state and corporate powers are uniquely interconnected within this practice (Whyte 2014), this method also helps to evaluate the state-corporate crime framework (see Davies and Wyatt 2021). As Bisschop et al. (2018) and Bradshaw (2015) illustrate, the state-corporate crime theory is permanently being adjusted through the analysis of different case studies.

## Revolving Doors in the Spanish Fossil Fuel Industry

### Background

The RDs between public office and the electric and fossil fuel sector are widespread around Europe. There are cases reported in the United Kingdom, France, Sweden, Norway, Italy, Portugal, and Spain, among others (see Greens/EFA 2018; Bautista 2018a, b). As Greens/EFA (2018) and Fossil Free Politics (2021) report, the RDs between public officials and the fossil fuel industry impact the European climate and energy policymaking agenda, deterring the transition towards renewable energy sources.

Spain regulates the RDs phenomenon under Law 3/2015, of March 30, regulating the exercise of the senior position of the General State Administration.^1^ It is an administrative law that, on the one hand, prescribes a cooling-off period of two years for both Ministers and members of the government and civil servants, advisors, and other public officials who want to move to the private sector (Bautista et al. 2018a, b). On the other hand, it establishes the Conflict of Interests Office (OCI) to evaluate each case and grant permission to transfer from public to private service if any conflict of interests is not foreseen (see Bautista et al. 2018a, b). Yet, these procedures are not successfully preventing the movement from public office to private companies due to the limited regulation of conflicts of interest and the cooling-off period (see Cocciolo 2020). As Reyes (2018) reports, between 2015 and 2017, 137 public officials got permission to occupy positions in private corporations, often ignoring the 2-year cooling-off period (Reyes 2018).

### ‘Revolving Out’

The RDs in the fossil fuel sector comprise of a long list of politicians and public officials working for electric companies after leaving public office (Reyes 2018). As Reyes (2018) reports, since the return of democracy in 1977, almost half of the Ministers started to work in private companies after leaving public office—most of them as directors, board members, or advisors of Endesa, Iberdrola, Naturgy (Gas Natural), Enagás, Repsol, and Red Eléctrica de España (REE), which are the dominant, transnational, and most pollutant fossil fuel and energy companies in Spain (see Buades 2021).

The revolving out mainly affects the leading political parties of the last decades and the most influential politicians (see Bautista et al. 2018a, b; Reyes 2018). In this vein, the political parties mainly involved are the Peoples’ Party (PP) and the Spanish Socialist Workers’ Party (PSOE, in its Spanish acronym). Regarding the public officials engaging in revolving out, it mostly involves high-ranking politicians (Greens/EFA 2018). For example, the Presidents of Spain, who liberalized the Spanish public electric assets, along with Vice-Presidents, Spokesmen, Ministers, Secretaries of State, Subsecretaries, and Advisors (see Bautista et al. 2018a, b; Reyes 2018; Spanish Revolution 2019; El Salto 2021).

While some public officials can work for a private company once they leave public office due to their expertise and academic background, most of the individuals involved in RDs have no previous experience in the sector (Greens/EFA 2018). For instance, the ex-director of the Guardia Civil, the oldest law enforcement agency in Spain, has no expertise related to the electric sector (Bautista et al. 2018a, b). Despite the lack of proficiency, in 2018, he had an annual remuneration of €156.000 as advisor of REE (ABC 2018). According to ABC (2018), the ex-director increased his salary by 30%, doubling the retribution of the president of the government. This is not an isolated example. As Reyes (2018) reports, the total remuneration of the 58 former Ministers that were board members of the Spanish-leading companies in 2016 was 18 million euros annually.

Spain also has cases revolving out less publicly lauded but equally harmful. To name a few, the Vice-President of Spain, previously State’s Attorney, who supervised the cuts to subsidies for the development of renewable energies in 2013, got a post-government job in a law firm in 2019, without a cooling-off period (Vélez 2019). As Vélez (2019) reports, the law firm is part of the advisory team of some private corporations suing Spain for the cuts to renewable energy subsidies (e.g., OperaFund or Schwab Holdings). Moreover, the Vice-President of the European Investment Bank (EIB), who supervised the loans of the Bank of Spain to Iberdrola, got a job at the Administrative Board of the company only three months after leaving the job at the EIB in 2021 (El País 2021). Due to the conflict of interests that can arise from it, at the time of writing, the case is being investigated by the European Ombudsman (see El País 2021).

The lack of experience, the high remunerations, and the previous connections with the private companies lead to questioning whether individuals involved in revolving out are selling their access or expertise. According to Reyes (2018) and Wilks-Heeg (2015), this practice creates fertile ground for corruption and influence-peddling.

### ‘Revolving In’

The Secretary of State for Economy and Energy was ‘revolved out’ in 2009 when appointed as advisor in Endesa, but in 2011 ‘revolved in’ to the Minister of Economy (Bautista et al. 2018b). However, of particular interest here are the lobby groups, consultancies, and corporations involved in policymaking. As mentioned above and as Wilks-Heeg (2015) defines, these cases do not need the actual move into the administration.

Fossil Free Politics (2021) found that, at the EU level, fossil fuel corporations and their lobbies are influencing energy policymaking not only through traditional RDs movements but also by being involved in policy development. For instance, the Spanish Confederation of Business Organizations (CEOE), the leading business lobbying group in Spain, has been in direct contact with the Spanish Government to elaborate regulation and policy documents (ENCO 2021). As the European Network of Corporate Observatories (ENCO) reports, members of this lobby include Enagás, Endesa, Iberdrola, Naturgy, and Repsol (ENCO 2021). Along with the oil and electric companies, the group includes large private consultancies like KPMG, Deloitte, PWC, and EY. These consultancies not only have developed regulation in Spain, but they are also part of its implementation (ENCO 2021), discussed below.

### Legislating in Favor of Public Interest?

It is not surprising that governments and electric companies work together as energy is a basic need for society. However, its regulation should defend the public interest instead of satisfying individual and industry interests (see Cortese 2011). This section provides examples of legislation developed by politicians now working for the companies that ultimately benefitted from these laws; and regulations captured by the industries they regulate. It demonstrates the symbiosis between the public and private sectors and the potential harm this practice entails.

Law 54/1997 of 27 November 1997 on the Electricity Sector^2^

The president of REE claimed that for the electricity sector “it is the mother of all laws” (Bautista et al. 2018b: 35). Law 54/1997 liberalized the electricity sector by setting two private companies to assume the previously public functions (Bautista et al. 2018b).

As Ortega-Almón et al. (2003) explains, there have been two privatization periods in Spain. In the first one, from 1985 to 1996 and called the ‘silent privatization’ (Ortega-Almón et al. 2003: 6), the PSOE started a State disinvestment process in public companies. But when the PP came to power in 1996, they initiated the privatization process of these companies (Ortega-Almón et al. 2003). The Presidents of both governments, the first member of the PSOE and the second of the PP, ended up as Board members of Gas Natural and external advisor in Endesa, respectively, after leaving public office (Reyes 2018).

As Bautista et al. (2018b) explain, the PP finished what was started by the PSOE by approving Law 54/1997. According to the preamble of Law 54/1997:

“This Law has […] as a basic purpose to establish the regulation of the electricity sector, with the triple and traditional objective of guaranteeing the electricity supply, guaranteeing the quality of said supply and guaranteeing that it is carried out at the lowest possible cost, all without forgetting the protection of the environment […] The national electricity system is no longer a public service of State ownership […] and its functions are assumed by two commercial and private companies.”

As Bacon (1995) claims, at the beginning of the 1980s, many countries had a public-owned electricity supply industry. However, within a decade, many European countries started changing the structure and ownership of the electricity sector, which meant the privatization and the splitting of the single previous firm into several firms. Spain established a privately owned asset but without achieving the free-competition structure. Despite the liberalization of the electricity market through Law 54/1997, the regulation failed to separate the operations of the electricity supply chain. “Traditional utilities have constituted clusters allowing them to cover all the economic activities from generation to the retailing”, the effect being that, “[i]n 2013, the traditional utilities coordinated in UNESA [the association of the five bigger traditional utilities, now called AELEC] comprised: 72% of generation, 98% of the distribution and 80% of the retailing of electricity in the country” (Capellán-Pérez et al. 2018: 219). Additionally, Capellán-Pérez et al. (2018) note that the regulation was unable to control the costs given that, in the Spanish system, transportation and distribution are self-audited by the transmission system operator (REE) and the power distribution utilities (the same integrating UNESA), and is allowed by the State.

Some of the members of the 1996–2000 government behind this law have been working for or had fraudulent business relations with electric companies after leaving public office. To name a few, the President of the State, who after his term worked as an external advisor for Endesa (Reyes 2018). In addition, the Second Vice-President and Minister of Economy got a turnover of 25.8 million euros through false advertising contracts with this company (Bautista et al. 2018b). And, the Minister of Environment of that time, who since 2014 has been a Board member of Enagás (Gonzalez-Navarro 2018; Reyes 2018).

According to Bacon (1995), having a monopolistic or oligopolistic system after privatization endangers competition. As mentioned above, this is the case of the electricity companies in Spain, which continued influencing the market. The structure of the Spanish electricity sector is a direct consequence of this movement.

Royal Decree 1432/2002, of December 27, which establishes the methodology for the approval or modification of the average or reference electricity tariff and modifies some articles of Royal Decree 2017/1997^3^

In 2002, the Minister of Economy and Second Vice-President, mentioned in the previous law, designed the Royal Decree 1432/2002 to set a limit on the price of electricity and, consequently, to stop inflation (Bautista et al. 2018b). The electric companies complained that the cost of electricity was not the one established by law and claimed that if they could not set the prices they wanted, they could not make the necessary investments in infrastructure. Since then, an extra cost was added to the electric bill to compensate the companies, known as ‘tariff deficit’ (Bautista et al. 2018b). It meant another increase in energy prices, even though the government promised that costs would fall after privatization (Ortega-Almón et al. 2003).

Like the law explained above, members of the second government of the People’s Party responsible for designing and approving this law went on to work for electric companies. In this case, the President, the Second Vice-president, and the Minister of Economy. Also, two of the Secretaries of Economy and Energy (Bautista et al. 2018b; Reyes 2018). One was President of REE from 2011 to 2018 (Bautista et al. 2018b; Reyes 2018), and the other became advisor of Endesa from 2009 to 2011 before serving as Minister of Economy (Bautista et al. 2018b), becoming a ‘revolving in’ door. Also, the Minister of Justice was named advisor of Iberdrola in 2012 (Bautista et al. 2018b; Reyes 2018). The General Director of Energy Policy and Mines holds positions at the Board of Directors of Endesa and REE (Bautista et al. 2018b). In addition, the Advisor of the Minister of Science and Technology was named advisor of Iberdrola years after leaving public office (Bautista et al. 2018b). The Minister of Foreign Affairs, who became a board member of Enagás along with the before mentioned Minister of Environment (Bautista et al. 2018b; Gonzalez-Navarro 2018); the Spokesman, appointed corporate CEO of Endesa in 2007, and the Secretary of State of Foreign Affairs, who in 2004 was appointed president of Iberdrola (Bautista et al. 2018b).

Law 24/2013, of 26 December, of the Electricity Sector^4^ and Royal Decree 900/2015, 9 October, which regulates the administrative, technical and economic conditions of the methods of supplying electricity with self-consumption and production with self-consumption^5^

Law 24/2013 derogated Law 54/1997 and reformed the electric sector by establishing the popularly known 'tax on the sun', which imposed payments on electricity self-consumption (typically generated through solar panels) (Bautista et al. 2018b), hindering and deterring Spanish citizens from choosing sustainable and renewable energy sources (Reyes 2018). In addition, subsidies were cut for renewables while maintaining fossil fuel subsidies which favored coal, *natural gas*, and nuclear energy (Reyes 2018). Later, through the Royal Decree 900/2015 the Spanish government implemented a tax that made solar energy users pay the electric companies to be connected to the electricity grid. Since 2018, the production of solar energy is being incentivized through the implementation of the Royal Decree-Law 15/2018, of 5 October, on urgent measures for energy transition and consumer protection,^6^ and the Royal Decree 244/2019, of 5 April, regulating the administrative, technical and economic conditions of the self-consumption of electric energy.^7^

Among the politicians behind these laws is the Cabinet Director of the Secretary of State of Energy. The Cabinet Director started as vice-secretary of REE only seven months after leaving public office (Bautista et al. 2018b). In addition, the General Secretary of Industry worked as an advisor for Enagás only two weeks after leaving public office (Bautista et al. 2018b). And the vice-president, a previous State attorney, started working for the law firm representing the companies suing Spain for the cuts on renewable energy subsidies right after leaving public office (Vélez 2019).

### The Recovery, Transformation and Resilience Plan (España Puede)

The Recovery, Transformation, and Resilience Plan for the Spanish Economy is the product of the economic recession following the COVID-19 pandemic (Presidency of the Government of Spain 2020). This Plan comes from the European Union's new recovery plan (Next Generation EU) which enables Spain and the other Member States to mobilize an unprecedented volume of investment to recover from the sanitary and economic crisis (Presidency of the Government of Spain 2020).

The Plan sets a series of goals aligned with achieving sustainable growth and creating jobs, accelerating the technological and digital transformation, favoring the creation and competitiveness of companies and industries, and protecting young people and the vulnerable. Of particular interest is the aim of having:

“A country committed to decarbonization, that invests in green infrastructure and that abandons fossil fuel energies in favor of a clean energy system, promoting new, safe and affordable developments, ensuring the sustainability of our production model, and fostering adaptation and resilience in the face of climate change”

(Presidency of the Government of Spain 2020: 10)

Even though the Plan promotes a transition towards a clean energy system, ENCO (2021) reports that it has been drafted by the same lobbying group ‘revolving in’. This includes the Spanish Confederation of Business Organizations (CEOE) and the large consultancies KPMG, Deloitte, PWC, and EY. As reported, such consultancies and their clients (Endesa or Repsol, for example) will also be part of the implementation of the recovery plan through hydrogen projects. These projects will continue with business as usual (ENCO 2021). Indeed, the big fossil fuel corporations applied with major projects to get the funds for the recovery assigned to Spain (ENCO 2021). As ENCO (2021: 2) claims, “Endesa, Naturgy and Iberdrola alone have proposed projects worth €53 billion, which if built would account for more than 70% of Spanish recovery funds”. Overall, their participation in the regulation and policy development in Spain and their participation in the implementation represents an enormous conflict of interests (ENCO 2021). Despite focusing on the Spanish case, ENCO (2021) also scrutinizes the role of lobby groups in the recovery plans developed in Portugal, Italy, and France, highlighting the overall ‘revolving in’ of fossil fuel interest groups in the EU policy development (see also Fossil Free Politics 2021).

As explored next, the regulation and policies influenced by the RDs have the potential to harm humans and environments alike. The case study brings to light the extent of the harms that can emanate from the RDs between the public sector and the fossil fuel industry and demonstrates how, in the pursuit of satisfying individual and industry interests at the expense of human and environmental well-being, the RD are inherently harmful.

## The ‘Revolving Doors’, a Legal Wrongdoing

### Social Harm

Law 54/1997 explained above highlights the social harms arising from the revolving doors phenomenon. As seen, this law liberalized the electric assets but without opening the market to free competition. According to Falcón-Pérez (2020), privatization led to a 70% increase in the electricity bills of Spanish citizens. Along with the increment of electricity bills, we also find the tariff deficit. As seen through the main reforms of the electricity sector, Spanish citizens are paying for the debt that the government generated to electric companies. As Reyes (2018) explains, this meant an increase in electricity prices during the 2008–2012 economic crisis, when consumer prices increased an average of 9.9% per year. Throughout 2021, Spanish citizens have experienced another increase in electricity bills because, according to the marginalist system, the price is determined by the most expensive electricity source (natural gas), hindering the economic recovery after the COVID-19 crisis. Following the scarcity of natural gas, prices skyrocketed. As reported, in October 2021, prices reached €204,09 per MWh, six times higher than October 2020 (OCU 2021).

At the core of the impacts of the RDs at a socio-economic level is energy poverty. Energy poverty is defined as “the absence of sufficient choice in accessing adequate, affordable, reliable, high-quality, safe and environmentally benign energy services to support economic and human development” (Reddy 2000: 44). As explained by González-Eguino (2015), it has an impact on millions of lives worldwide, with an estimated 1.3 million deaths per annum linked to indoor pollution. Along with the consequences on health, energy poverty fails in supporting human development by excluding people from choosing welfare (González-Eguino 2015). In this vein, energy poverty not only deprives access to essential services like heating, but it also goes against other fundamental rights such as education and health. Moreover, the RDs practice, along with other corrupt practices, undermines confidence in public institutions. This constitutes social harm because public institutions represent fundamental democratic values and, if delegitimized, democracy itself is endangered (see Cocciolo 2020). Then, the revolving doors have important implications for society.

### Environmental Harm

The RDs are also propitiating a reliance on fossil fuels. As seen throughout the cut on renewable energy subsidies while maintaining subsidies to fossil fuels, the tax on self-consumption, and a Recovery Plan drafted by the companies responsible for most of the GHG emissions, the use of renewable energy sources has been sabotaged. These policies damaged the profitability of transitioning towards renewable energy sources and resulted in disinvestment in renewable energies. As a result, the RDs practice causes climate change through climate policy (in)action (see Lynch et al. 2010). According to Kramer (2014), climate change is one of the forms of environmental crime that produces the broadest range of victims, described as an apocalyptic event and an ecological catastrophe.

Moreover, the world is now heading into an energy crisis. As Helman (2021) reports, in the United States, crude oil prices increased by 65%, natural gas has more than doubled its cost, and coal is up 400%. However, the energy crisis is hitting Europe more severely. According to Fleming (2021), the increasing demand for gas as economic activity recovers from the COVID-19 pandemic, the limited supplies leading to an increase in prices (at the time of writing, 600%), and an energy transition reliant on natural gas all fuels the current European energy crisis. As seen before, the Spanish Government favored the use of natural gas over renewable energy sources (Reyes 2018). Despite being cleaner than coal or oil, natural gas is still a fossil fuel and a pollutant, emitting large quantities of carbon dioxide and, most importantly, methane (ENCO 2021). Natural gas dependence not only leads to climate change promotion, but its scarcity also means that prices are likely to continue to soar, increasing social and economic instability.

Furthermore, as seen through the Recovery Plan, big oil and gas corporations are using hydrogen projects to access public funds and capture the development of new technologies. Hydrogen projects have the potential to perpetuate the use of fossil fuels instead of developing green hydrogen technologies (representing only 0.1% of the production) because grey hydrogen is predominant (80%) and generated using natural gas (ENCO 2021). In addition to grey hydrogen, given the decrease in demand for coal and oil following the carbon tax, fossil fuel companies are marketing the use of plastics (The Story of Plastic 2019). As reported, plastics present an opportunity for big oil corporations to keep up demand (The Story of Plastic 2019), leading to a whole new range of harms attributed to plastic pollution (see Smith and Brisman 2021). Thus, fossil fuels are finding a new form and place to flow through the economy in the form of plastics and hydrogen, damaging the climate and environment even further.

Harms to humans and the environment are connected, one reinforcing the other. As González-Eguino (2015) explains, energy poverty impacts health and frustrates environmental protection efforts. As we have seen, privatization increased the electricity price, which fueled energy poverty within Spanish society. At the beginning of 2021, in the middle of the COVID-19 pandemic and the storm Filomena, seven out of 100 Spaniards were estimated to be living in a situation of energy poverty (RTVE 2021) because the electricity bill increased an average of 18.4% in comparison to the same month last year (Europa Press 2021). Consequently, Spanish society relied on cheaper energy sources and technologies like butane gas, with demand increasing by 40% (Ara 2021). In this vein, the laws developed in Spain impact climate change by fostering the use of dirtier but more affordable energy sources and technologies. The increase in demand for fossil fuels contributes to climate change. Therefore, extreme weather events (like the storm Filomena) will happen more frequently. This will lead to generalized extreme temperatures further increasing electricity bills, and the need for more electricity (such as heating or air conditioning), conducing a vicious circle between energy poverty and climate change.

Agreeing with Michalowski and Kramer (2006), the research finds that when the political and economic powers intersect the extent of harm is magnified. As Bradshaw (2015) claims, and the case study illustrates, state and corporate actors are increasingly working toward the shared goals of privatization and private capital accumulation at the expense of human rights and environmental protection. However, within the RDs, the intersection of both powers is even more pervasive. The RDs erase the line that separates the regulator from the regulated. This feature builds on the state-corporate crime theory and concept with insights from the idea of regulatory capture (see Cortese 2011), discussed below.

## State Capture, State-Corporate Crime

As Whyte (2014) argues, the state-corporate crime theory allows us to bring the state back into the study of corporate crimes. As mentioned above, the state-corporate crime theory differentiates between initiated and facilitated, but these are limited if analyzing the role of the political economic structure in producing the harm (Whyte 2014; Bernat and Whyte 2017, 2020). As Whyte (2014: 238) describes, the terms initiated and facilitated imply that there have been ‘moments of rupture’. In this vein, the state-corporate crime theory limits the state-corporate wrongdoing to a moment of failure in the coercive capacity of the state (Whyte 2014). In other words, state-corporate crimes happen because the state either had an active involvement or failed in controlling the corporate crime. However, these do not fully capture the increasing connectedness of both powers in the neoliberal era. The exceedingly apparent relationship between the public and the private sector within the RDs highlights the need to go beyond these moments of rupture (Whyte 2014).

Deregulation is a term that ‘obscures’ its status as a crime (Bernat and Whyte 2020: 127). States play a central role in re-regulating in favor of the corporation (Tombs 2016a, b). As claimed by Whyte (2020: 111), “the state is the lifeblood of the corporation, from the point that it establishes the rules of incorporation”. Precisely, using the term ‘re-regulation’ brings to light the active role of the governments in creating a criminogenic structure (Tombs 2016b: 3). Since the function of states is not the defense of the public interest, public and private sectors cannot be seen as antagonistic elements because “corporations are given life by the state ensemble” (Bernat and Whyte 2020: 132). Because the state created the setting where state-corporate crimes exist, it needs a more prominent role. Therefore, the state-corporate crime theory urges a far-reaching understanding of the complex relationship between both powers.

As in previous case studies (see Lasslett 2014; Bradshaw 2015), the RDs in the fossil fuel industry in Spain serve to fine-tune the state-corporate crime theory. As found, the RDs are not simply a set of cases of individual wrongdoing, which could be associated with diverse moments of rupture, but a systemic problem across institutions and industries (Fossil Free Politics 2021). The RDs practice emanates from an architecture of power in which power remains within the same social elites, guaranteeing the protection of individual and industry interests. Thus, the RDs are a manifestation of the architectural design of the political economy as the origin of state-corporate crimes.

Within neoliberalism, states create a fertile ground for corporations, even if it damages the public interest. The opposite of regulating in favor of the public interest is regulatory capture (Levine and Forrence 1990; Cortese 2011). The concept of regulatory capture claims that legislation is established around the demands of industry interests (Cortese 2011). Thus, the industries subject to regulation use their powers to assure that the rules are favorable to their activities (Uche 2001; Cortese 2011). This dimension of regulatory capture is of particular interest when examining RDs movements (see Makkai and Braithwaite 1992).

In established democracies, “agenda-setting and policy design have increasingly been outsourced to professional consultancies, third-sector agencies, law, and accountancy firms and corporate sponsored think tanks” (Innes 2021: 1). As seen with the Recovery, Transformation, and Resilience Plan, the Spanish state externalized policy development to industry structures, erasing the already thin line separating both powers. It illustrates how the original concepts overlook the complex relationship between the state and corporations in which the state is paving the ground for the production of state-corporate crime.

Bradshaw (2015) argued that the RDs phenomenon falls within the scenario of state-facilitated corporate crime because governments pass laws that seem to facilitate corporate wrongdoing. RDs could also be classified as corporate-initiated state crime because corporations appear to manipulate governments into taking harmful actions. But, as this paper illustrates, it is not that the government fails in regulating the corporate activities or that corporations influence states to the detriment of the public interest. The RDs are an essential feature of the structure of contemporary capitalism, in which industries permeate public policymaking. The research asserts that the original theory requires a classification to reflect how, in some cases, state-corporate crimes are the product of the neoliberal structure itself. To this end, this paper proposes the concept of state-capture corporate crime to refer to these harms resulting from corporate state capture. It helps to build this structural criminogenic environment where RDs emerge and are normalized (Bradshaw 2015).

State capture expands on regulatory capture by referring to the systemic corruption in which the state’s role of policymaking is controlled by private corporate groups (Innes 2021). As Innes (2021) explains, state capture privileges the interests of certain social groups which, within neoliberalism, are large firms and multinational corporations. Consequently, the concept of state-capture corporate crime seeks to bring to light the structural dimension of state-corporate crimes within neoliberal economies. Because the RDs are not the product of a moment of rupture but a structural issue of corporate state capture (see Innes 2021), the case study demonstrates the need of widening the original concept to examine how corporate state capture enables state-corporate crime.

## Conclusion

The case study of the RDs in the fossil fuel industry in Spain brings to light the extent of the social and environmental harms that can emanate from this practice. As explored throughout the paper, the RDs are accountable for socio-economic instability and responsible for the climate policy inaction contributing to climate change on a global scale. Further, this paper emphasizes the need to go beyond the study of moments of rupture to understand the structural cause of state-corporate crimes. It refers to the architecture of power in which states grant corporations various privileges and infrastructural capabilities leading towards an unprecedented state-corporate symbiosis. The RDs examined in this article reflect the interconnectedness between the public and private sectors.

This paper encourages a broader conceptualization of the RDs and the state-corporate crime concepts. As found, it would help to capture the public–private criminogenic structure where state-corporate crimes emerge. On the one hand, while traditionally the RDs referred to actual movements of individuals between public office and the private sector, this research brings to light a new dimension of revolving in without moving. It illustrates how, within neoliberalism, the private sector permeated public office and the policy-making process to engage in practices of self-regulation, thereby damaging humans, non-human species, and environments alike. State capture reflects this extreme connectedness where the line differentiating the two sectors has been erased. The concept of state-capture corporate crime stresses that state-corporate crimes are the product of modern capitalist societies. In light of the above, the article concludes that it is crucial to get an in-depth understanding of the nature, extent, and impacts of the RDs across neoliberal societies and industries to build on the state-corporate crime theory and to expose the harms arising from this practice, which otherwise will remain invisible.

## Footnotes

1 Law 3/2015, of March 30, regulating the exercise of the senior position of the General State Administration (BOE, no. 77, 3–31-2015, pages 27,259 to 27,277).

2 Law 54/1997, of November 27, on the Electricity Sector (BOE, no. 285, 11–28-1997, pages 35,097 to 35,126).

3 Royal Decree 1432/2002, which establishes the methodology for the approval or modification of the average or reference electricity tariff and modifies some articles of Royal Decree 2017/1997, of December 26, by which it is organized and regulated the procedure for the settlement of the costs of transport, distribution and commercialization at tariff, of the permanent costs of the system and of the costs of diversification and security of supply (BOE, no. 313, 12–31-2002, pages 46,333 to 46,338).

4 Law 24/2013, of December 26, of the Electricity Sector (BOE, no. 310, of 12/27/2013, pages 105,198 to 105,294).

5 Royal Decree 900/2015, of October 9, which regulates the administrative, technical and economic conditions of the methods of supplying electricity with self-consumption and production with self-consumption (BOE, no. 243, 10–10-2015, pp. 94,874 to 94,917).

6 Royal Decree-Law 15/2018, of October 5, on urgent measures for the energy transition and consumer protection (BOE, no. 242, 6–10-2018, pages 97,430 to 97,467).

7 Royal Decree 244/2019, of April 5, which regulates the administrative, technical and economic conditions for self-consumption of electric energy (BOE, no. 83, 6–4-2019, pages 35,674 to 35,719).
